# Training the Gatekeepers of Research Integrity: Enhancing peer review and editorial Process excellence by simulated training programs

**DOI:** 10.12669/pjms.41.5.12262

**Published:** 2025-05

**Authors:** Sadia Yaseen

**Affiliations:** 1 Dr. Sadia Yaseen Assistant Professor Medical Education, Rashid Latif Medical College, Lahore, Pakistan. CEO, MedicalEducation.ORG Email: sadiayaseendme@gmail.com

**Keywords:** Simulated Training program, Peer review, Research integrity, Editorial process

Peer review and editorial processes are embedded in checkpoints of the research process to ensure the legitimacy and quality of scholarly work and research integrity. Despite this fact, many editorial staff and peer reviewers are frequently deficient in formal training. Considerable discussion has been initiated by well-cited editorials which either endorsed or criticized the peer review system. This editorial emphasizes the need for a simulated training program for editorial staff and peer reviewers. It also highlights the significance, challenges and opportunities regarding this program.

Peer review fundamentally requires a robust knowledge of relevant research methodologies, ethical considerations and critical analysis along with expertise in the subject matter. A well-structured peer review can considerably enhance the quality of published research. However, many peer reviewers enter their role with little or no formal training, just drawing upon their experience as authors or readers of manuscripts. The training aimed at producing high-quality peer reviewers can potentially enhance the overall quality of published literature.^1^

The research identified a gap in training editorial staff and peer reviewers. This self-taught process is ineffective and lacks consistency.^2^ A significant issue is that the editorial process and peer reviews have been largely least effective in identifying lapses in scientific rigor e.g., missing statistics, data fabrication, or detection of bias. Many times, reviewers fail to provide constructive feedback or neglect critical flaws.^3^ So, the editors must not expect reviewers to identify statistical glitches and major errors particularly associated with the study context. Short training courses may impact such error detections.^4^

A simulated training program appeared to be a solution with great potential to tackle this research gap is a pedagogical approach to prepare them with the required skill and knowledge to perform a thorough and well-structured peer review and editorial process through practical, hands-on learning that simulates real-life scenarios. This could include mock manuscripts, mock editorial triage, role-playing exercises, annotated reviews, review scoring systems, online simulation tools, reviewing published papers, workshops and seminars and feedback sessions. Such programs can enhance the critical evaluation skills of peer reviewers and editorial staff.

Moreover, peer reviewers and editorial staff must be trained to use Artificial intelligence ethically to lessen the burden. Many artificial intelligence software dedicatedly available for this purpose like stat checker, Penelope AI and stat reviewer to improve the detection of statistical misconduct.^5^ These tools are developed to support editors, peer reviewers and publishers at different stages of peer review process. These tools assist editorial staff and peer reviewers in evaluating the quality of manuscripts, understanding the complexity of manuscripts and even sometimes detecting AI generated plagiarism. Literature reveals the potential of these tools that using such tools reduce editorial burden more than 70% for reviewer allocation.^6^

The curriculum of such courses must include content expertise, critical evaluation skills, proficiency in written communication skills and comprehending the ins and outs of the science of publication.^7^ The training programs for editors must entail the role of editors, understanding editing and manuscript publishing, skills in identifying potential reviewers, Committee of publication ethics (COPE) and international committee of medical journals editors (ICMJE) guidelines and the editorial process post-acceptance manuscript.^8^

In our context in Pakistan, such courses are direly required but it’s not smooth sailing due to various challenges. In 2011, World Health Organization (WHO) EMRO (Eastern Mediterranean region organization) countries arranged a training session for editors all around the world conducted at Sheraz University of Medical Sciences, Islamic Republic of Iran. As a mandatory requirement of WHO, Mr. Jawaid on his return to Pakistan conducted various sessions for the training of editors and peer reviewers. In 2015, it was suggested to start a master’s in medical journalism. But the idea fell through and was put on the back burner. One of the biggest challenges was to sensitize the stakeholders. Another obstacle was that most of the editors were working on an honorary charge without any contract, so they were unsure of the fate of their role even after getting trained. The successful widespread initiation of such training programs in the country requires a robust advocacy and sensitization campaign.^9^ A certificate course in medical editing at the University of Health Sciences was initiated later. Now recently, University of Health Sciences initiated a postgraduate diploma in Medical Journalism and the program is overseen by Prof Nadia Naseem, editor-in-chief of BioMedica. This program fulfils all the requirements of simulated training.

In conclusion, editors and peer reviewers are gatekeepers of the quality of manuscripts and research integrity. Their training will ensure quality critical evaluation of manuscripts along with content expertise and a well-structured robust editorial process leading to enhanced standards of scholarly work and thorough and robust publication. Simulated training programs are widely required in our context. To overcome the challenges the stakeholders must be on board and must recognize the significance of the program. Journals should start sensitizing their reviewer about formal training.

**Fig.1:**
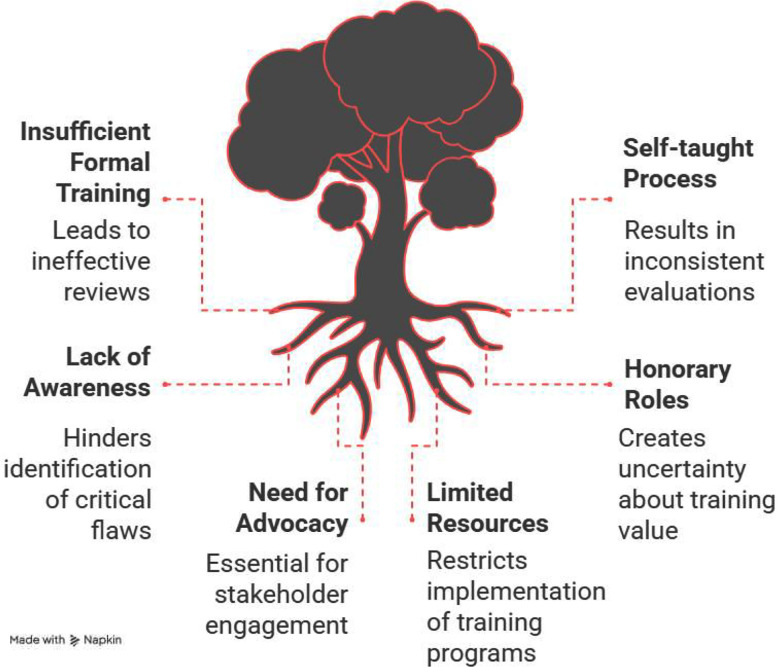
Lack of Training for Peer Reviewers and Editors and its impact.

## References

[1] Buser JM, Morris KL, Dzomeku VM, Endale T, Smith YR, August E (2023). Lessons learnt from a scientific peer-review training programme designed to support research capacity and professional development in a global community. BMJ Glob Health.

[2] Proctor DM, Dada N, Serquiña A, Willett JLE (2023). Problems with peer review shine a light on gaps in scientific training. mBio.

[3] Baxt WG, Waeckerle JF, Berlin JA, Callaham ML (1998). Who reviews the reviewers?Feasibility of using a fictitious manuscript to evaluate peer reviewer performance. Ann Emerg Med.

[4] Schroter S, Black N, Evans S, Godlee F, Osorio L, Smith R (2008). What errors do peer reviewers detect, and does training improve their ability to detect them?. J R Soc Med.

[5] Checco A, Bracciale L, Loreti P, Pinfield S, Bianchi G (2021). AI-assisted peer review. Humanit Soc Sci Commun.

[6] Shah FA, Jawaid SA (2025). The inevitable future of peer review:Human and AI integrated peer review system. Pak J Med Sci.

[7] Lyons-Warren AM, Aamodt WW, Pieper KM, Strowd RE (2024). A structured, journal-led peer-review mentoring program enhances peer review training. Res Integr Peer Rev.

[8] Reflecting on the Communications Biology Editor Training Program (2024). Commun Biol.

[9] Jawaid SA, Jawaid M (2017). Professional competencies required for Editors of Biomedical Journals. Pak J Med Sci.

